# What does static electricity has to do with schizophrenia?

**DOI:** 10.1192/j.eurpsy.2021.2123

**Published:** 2021-08-13

**Authors:** A. Paraschakis

**Affiliations:** Psychiatric Hospital Of Attica “dafni”, Department of General Adult Psychiatry, Athens, Greece

**Keywords:** paranoid, psychopathology, schizophrénia, Delusion

## Abstract

**Introduction:**

The suspiciousness of a paranoid patient could reach extremes.

**Objectives:**

To present the lessons learned after an interview with a similar patient.

**Methods:**

Case report.

**Results:**

Fifty-two year old woman suffering from paranoid schizophrenia (F20.0). Symptoms almost identical to her previous (two) hospitalizations: delusions of thought reading and of being surveilled by electronic equipment in her house/neighborhood. This time, though, she was additionally convinced of being the object of medical experiments and of being electronically surveilled even within the ward. Treatment: risperidone 12mg/day, lorazepam 3.75mg/day, biperiden 2mg/day. Three weeks after admission, the author noted a slight tremor in her hands (most certainly of extrapyramidal origin). I asked her to place both hands in front of her, fingers wide open, to assess it better. The patient followed with the fingers attached, though. Consequently, I approached my hands to hers -to show how it should be done correctly-, touching them lightly. Then, a spark was generated between our hands. Evidently, it was an electrostatic discharge (I was wearing a wool sweater that day; static electricity could easily accumulate on wool). She became outraged: “what kind of experiments are you doing to me?”, “what electronic devices are you using?”, “this is the proof of what I have been constantly saying”.

**Conclusions:**

The symptoms of psychotic relapses could evolve over time. A clinician should refrain from any strictly unnecessary physical contact with an exceedingly paranoid patient, particularly when the latter claims that is the object of “medical experiments”. The elaborative “ability” of such patients could be, simply, astounding.

**Disclosure:**

No significant relationships.

